# Coenzyme Q10 Supplementation in Statin Treated Patients: A Double-Blinded Randomized Placebo-Controlled Trial

**DOI:** 10.3390/antiox11091698

**Published:** 2022-08-29

**Authors:** Tine L. Dohlmann, Anja B. Kuhlman, Thomas Morville, Maria Dahl, Magnus Asping, Patrick Orlando, Sonia Silvestri, Luca Tiano, Jørn W. Helge, Flemming Dela, Steen Larsen

**Affiliations:** 1 Xlab, Center for Healthy Aging, Department of Biomedical Sciences, Faculty of Health Sciences, University of Copenhagen, 1165 Copenhagen, Denmark; 2 Department of Life and Environmental Sciences, Polytechnic University of Marche, 60121 Ancona, Italy; 3 Department of Geriatrics, Bispebjerg University Hospital, 2400 Copenhagen, Denmark; 4 Clinical Research Centre, Medical University of Bialystok, 15-089 Bialystok, Poland

**Keywords:** mitochondria, skeletal muscle, reactive oxygen species, HMG-CoA reductase inhibitor, myopathy, diet supplementation, antioxidant

## Abstract

Myalgia and new-onset of type 2 diabetes have been associated with statin treatment, which both could be linked to reduced coenzyme Q10 (CoQ10) in skeletal muscle and impaired mitochondrial function. Supplementation with CoQ10 focusing on levels of CoQ10 in skeletal muscle and mitochondrial function has not been investigated in patients treated with statins. To investigate whether concomitant administration of CoQ10 with statins increases the muscle CoQ10 levels and improves the mitochondrial function, and if changes in muscle CoQ10 levels correlate with changes in the intensity of myalgia. 37 men and women in simvastatin therapy with and without myalgia were randomized to receive 400 mg CoQ10 daily or matched placebo tablets for eight weeks. Muscle CoQ10 levels, mitochondrial respiratory capacity, mitochondrial content (using citrate synthase activity as a biomarker), and production of reactive oxygen species were measured before and after CoQ10 supplementation, and intensity of myalgia was determined using the 10 cm visual analogue scale. Muscle CoQ10 content and mitochondrial function were unaltered by CoQ10 supplementation. Individual changes in muscle CoQ10 levels were not correlated with changes in intensity of myalgia. CoQ10 supplementation had no effect on muscle CoQ10 levels or mitochondrial function and did not affect symptoms of myalgia.

## 1. Introduction

Statins (HMG-CoA reductase inhibitors) are the first choice for lipid lowering therapy used in the prevention of cardiovascular diseases. In Denmark, more than 20% of the population above 40 years [1] use statins, and with a continuous broadening of the guidelines for prescribing statins [2], the number of individuals eligible for statin treatment has continued to grow [3]. Although generally well tolerated, statins are commonly associated with muscular side effects that range from mild muscle pain (myalgia) to the severe muscle dissolution syndrome, rhabdomyolysis [4] and it has also been reported that new onset of type 2 diabetes is increased with statin treatment [5]. Statins are prescribed to almost all patients with type 2 diabetes in developed countries. Muscular side effects can interfere with the ability to perform daily activities and reduce the habitual physical level, and represents the most common cause of statin discontinuation [6]. Thus, researchers and clinicians have searched for ways to alleviate symptoms of statin-induced myalgia [7,8].

The etiology of statin-induced myalgia is not fully understood, but several studies have suggested a key role for depleted muscle coenzyme Q10 (CoQ10, also known as ubiquinone) levels [9,10,11] and impaired mitochondrial function [12,13,14]. CoQ10 is a vitamin-like molecule partly supplied from the diet but, like cholesterol, the majority is synthesized endogenously by the mevalonate pathway [15], which is inhibited by statins [16]. In the mitochondria, CoQ10 function as an essential electron carrier in the electron transfer system, and thus a void of muscle CoQ10 may impair the mitochondrial respiratory function and increase the production of reactive oxygen species (ROS) [17]. Mitochondrial respiratory dysfunction and elevated ROS production have previously been associated with nociceptor activation and the development of pain [18,19]. Thus, the association between statins, depleted muscle CoQ10, mitochondrial dysfunction, and myalgia is biologically plausible, although not confirmed in the literature.

Previous studies have investigated if oral CoQ10 supplementation alleviate the symptoms of statin-induced myalgia, but with mixed results, as some report no effects of CoQ10 supplementation [20,21,22,23,24] and others report diminished symptoms of myalgia [25,26]. None of the studies investigated if CoQ10 supplementation increased the muscle levels of CoQ10, and therefore it is unknown if oral CoQ10 supplementation increases muscle CoQ10 levels, and if changes in muscle CoQ10 levels affect the mitochondrial function and intensity of statin-induced myalgia. CoQ10 is widely consumed for several indications, and the National Health Industry Survey reported that 1.3% of the adults in the United States used CoQ10 supplements in 2012 [27]. The rationale for recommending statin users oral CoQ10 supplementation is an ongoing discussion among clinicians and laymen in both the scientific literature [28,29,30,31,32] and in the media [33,34], and there is still a void of scientific evidence supporting beneficial effects of CoQ10 supplementation for statin users. Therefore, we conducted a randomized, double-blinded, placebo-controlled trial to investigate whether supplementing statin users with CoQ10 increases the muscle CoQ10 levels and improves the mitochondrial function, and whether individual changes in muscle CoQ10 levels are correlated with individual changes in the intensity of myalgia.

## 2. Materials and Methods

This study is a randomized controlled trial and is part of the interdisciplinary “Living with Statins” (LIFESTAT) study [35]. The study is approved by the local ethics committee, Frederiksberg, Copenhagen (protocol number: H-2-2013-164) and the study’s a priori defined outcomes and rationale is defined at ClinicalTrials.gov (Identifier: NCT02255682). The a priori defined primary endpoint of the LIFESTAT-study is change in myalgia measured by the Visual Analogue Scale (VAS), and a priori secondary endpoints include changes in respiratory fitness (VO_2max_), muscle strength, glucose metabolism, and mitochondrial function. The overall study design and some of the study’s endpoints [36] and exploratory sub-analyses [37] have previously been published. This study investigates whether CoQ10 supplementation increases muscle CoQ10 levels and improves mitochondrial function in statin users, and whether statin users who report mild to moderate myalgia have alleviated intensity of myalgia after CoQ10 supplementation, which is correlated with changes in muscle CoQ10 levels. To address mitochondrial function, we completed detailed measurements of mitochondrial respiratory capacity, production of ROS, and mitochondrial content using citrate synthase (CS) activity as a biomarker. Exploratory analyses of apoptotic signaling are available in supplemental material. The study was conducted in accordance with the Helsinki declaration, and all subjects signed a written consent form before enrolling in the study.

### 2.1. Recruitment

**Figure 1:**
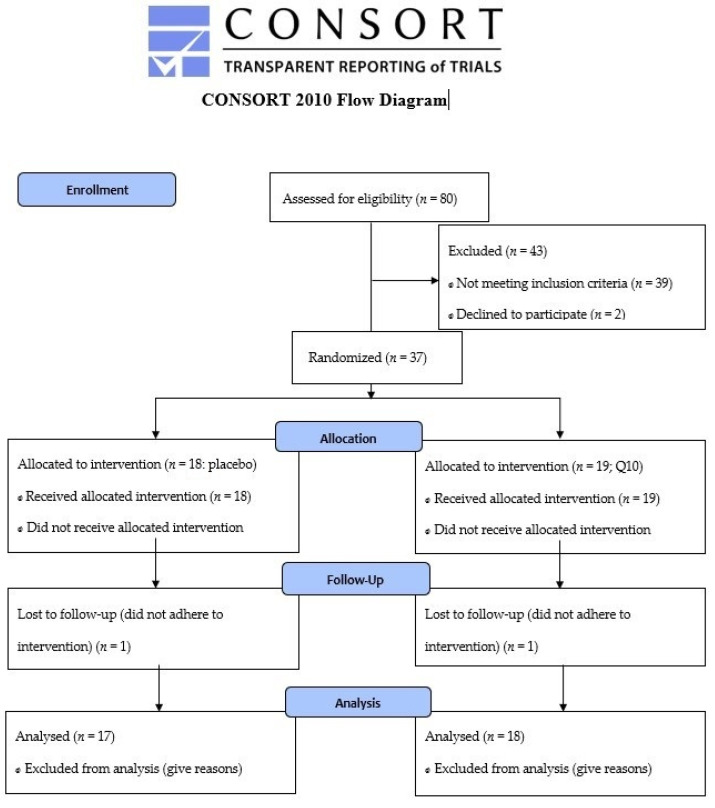
CONSORT 2010 Flow Diagram.

Patients in primary prevention with simvastatin minimum 40 mg daily, who met the inclusion criteria (age 40–70 years, body mass index 25–35 kg∙m^−2^), were invited for a personal interview where they described their history of statin use and, if any, symptoms of muscle pain. A flow diagram for the enrollment process is provided in Figure 1. Patients were recruited via advertisement in local newspapers and from their general practitioners. Any history of diseases (diabetes mellitus, arrhythmia, ischemic heart disease, musculoskeletal disorders), mental disorders that interfere with the ability to understand, or use of medication that could influence the study outcomes (medication that interacts with simvastatin or interferes with physical testing, e.g. β-blockers) led to exclusion. Informed consent was obtained from all subjects involved in the study.

### 2.2. Protocol

Study participants were randomly allocated in blocks of 2 (using randomizer.org) to consume either 2 × 200 mg CoQ10 or matched placebo capsules (soybean oil, similar size and colour) daily with a meal, for 8 weeks (PharmaNord Aps, Vejle, Denmark). Both participants and researchers were blinded and the randomization procedure was performed by the department secretary, who gave participants a bag of tablets for the whole intervention, at inclusion. The participants received exactly the number of tablets needed for the whole intervention period (counted by two research leaders) and were asked to contact us in case they needed more tablets and to return any remaining tablets when reporting to the laboratory for the final day of testing (one participant requested more tablets and none had remaining tablets to return). During the final days of the supplementation period, the participants were asked how the supplementation period had been and to remember to ingest the final tablets correctly (the final dose was to be ingested with a meal the night before obtaining muscle and plasma samples). Before and after the intervention, subjects reported to the laboratory after an overnight fast for testing. A detailed description of the protocol and biometric tests and blood sampling procedures in this study was presented previously [36]. Muscle biopsies were obtained from the vastus lateralis muscle with the Bergström needle technique modified for suction. A part of the muscle tissue was snap frozen in liquid nitrogen for western blotting of pro- and antiapoptotic proteins (online material), and approximately 40–50 mg of the muscle tissue was placed in cold biopsy preservation solution “Biops” (100 mM CaK_2_EGTA, 100 mM K_2_EGTA, 5.77 mMNa_2_ATP, 6.56 mM MgCl_2_ · 6H_2_O, 20 mM Taurine, 15 mM Na_2_Phospho-creatine, 20 mM Imidazole, 0.5 mM DTT, 50 mM MES) for measurements of mitochondrial respiration and Buffer X (60 mM K-Mes, 35 mM KCl, 7.23 mM K_2_EGTA, 2.77 mM CaK_2_EGTA, 20 mM Immidazole, 0.5 mM DTT, 20 mM Taurine, 5.7 mM Na_2_ATP, 15 mM Na_2_Phospho Creatine, 6.56 mM MgCl_2_·6H_2_O, H_2_O) for measurement of hydrogen peroxide (H_2_O_2_) production. H_2_O_2_ is measured as a proxy for ROS, as ROS produced by mitochondria is immediately converted to H_2_O_2_ by mitochondrial superoxide dismutase (MnSOD) [38]. The intensity of myalgia was scored using a 10 cm VAS. The introduction to VAS was standardized to ensure all subjects received the same information prior to scoring the intensity of myalgia.

### 2.3. Mitochondrial Respiration 

The muscle biopsy was dissected on ice and connective tissue was removed before permeabilization with saponin as previously described [39,40]. Mitochondrial respiration was measured using high resolution respirometry (Oroboros Instruments, Innsbruck, Austria) with hyperoxygenation [41]. The substrate-inhibitor (SUIT) protocols were as follows: Protocol I measured maximal complex I, complex I+II linked respiration and maximal uncoupled respiration using the following substrate combinations: malate (2 mM), glutamate (10 mM) and pyruvate (5 mM), ADP (5 mM) and MgCl_2_ (3 mM), cytochrome C (10 μM), succinate (10 mM), FCCP titration (0.25 μM steps until respiration declines). Protocol II investigated maximal complex I and complex II respiration: malate (2 mM), glutamate (10 mM) and pyruvate (5 mM), ADP (5 mM) and MgCl (3 mM), cytochrome C (10 μM), rotenone (0.5 µM), succinate (10 mM).

### 2.4. Production of Reactive Oxygen Species (Hydrogen Peroxide (H_2_O_2_))

H_2_O_2_ production and mitochondrial respiration were measured simultaneously with Oroboros Oxygraph-2-Flouroscence LED2-Module (Oroboros Instruments, Innsbruck, Austria) using a Fluorescence-Sensor Green (525 nm). The method is described in detail elsewhere [39]. In brief, all measurements were performed in Buffer Z (1 mM EGTA, 5 mM MgCl_2·_6H_2_O, 105 mM K-Mes, 30 mM KCl, 10 mM KH_2_PO_4,_ 5 mg·mL^−1^ BSA) with hyperoxygenation. Amplex Red (50 µM), superoxide dismutase (90 U·mL^−1^), blebbistatin (25 µM), and horse radish peroxidase (12 U·mL^−1^) were added before any substrates for mitochondrial respiration. Calibration standards of 40 µM H_2_O_2_ were prepared fresh every day. The SUIT protocol was: malate 5 mM and pyruvate 5 mM, succinate (3 titrations: 1 mM, 3 mM, 10 mM), ADP (5 mM) and MgCl_2_ (3 mM), myxothiazol (5 µM). All titrations were interspersed with titration of 0.1 µM H_2_O_2_ calibration standard to correct for background drift of H_2_O_2_.

### 2.5. Determination of Muscle CoQ10 Concentration and Citrate Synthase Activity

Intramuscular concentration of CoQ10 were quantified by high-formance liquid chromatography (HPLC)-UV (Shimadzu, Kyoto, Japan) as described elsewhere [42]. In brief, 40–50 mg of frozen muscle tissue was homogenized in butylated hydroxy toluene and ethanol and hexane were added. The samples were centrifuged, and the hexane phase was transferred to another vial, and the procedure was repeated. Before analysis, the samples were resuspended in mobile phase. Citrate Synthase (CS) activity in muscle tissue was measured by spectrophotometry (Cobas 6000, C 501, Roche Diagnostics, Mannheim, Germany), as previously described [43].

### 2.6. Determination of Plasma CoQ10 Concentration

Plasma CoQ10 content and its oxidative status were assayed as described by Orlando et al. [44]. Briefly, blood was drawn into heparinized vacutainers and immediately centrifuged, and plasma was stored at −80 °C until analysis. Hence, 50 µL heparinized plasma was extracted with 250 µL isopropanol and mixed vigorously by vortex. After centrifugation at 20,900× *g* at 4 °C for 2 min, 40 µL supernatant was injected into a HPLC with electro-chemical detector (ECD) (Shiseido Co. Ltd., Tokyo, Japan) and characterized by a pre-separation concentrating column and a post-separation reducing column (Shiseido CQR) able to reduce eluted ubiquinone.

## 3. Materials and Methods

CoQ10 (100 mg capsules) and matched placebo capsules were donated by Pharma Nord Aps (Vejle, Denmark).

### Statistics

The restrictive assumptions, normality, and equal variance were checked before the statistical analysis was conducted. Comparisons between groups were made by two-way ANOVA for repeated measures and Holm–Sidak for post hoc analyses. Data that were not normally distributed were log transformed before analysis. Pearson correlation coefficient was used to measure linear correlations. Statistical analyses were performed with SigmaPlot 13.0 (Systat Software, San Jose, CA, USA). Graphs were created with Graph Pad Prism 7 (La Jolla, CA, USA). Data are presented as mean ± SEM. Statistical significance was set at *p* < 0.05. Before initiation of the study, a power calculation was performed. Since there are no data available regarding CoQ10 supplementation and skeletal muscle CoQ10 content in statin treated patients, we could not use this in our calculation. We have therefore used data from studies investigating plasma changes after CoQ10 supplementation and mitochondrial measurements from studies previously conducted in our laboratory. These calculations showed that n = 15 in each group would be sufficient.

## 4. Results

### 4.1. Characteristics

Some characteristics from this cohort have previously been reported [36] and are given in Table 1. One subject in each intervention group did not adhere to the intervention and was excluded, leaving 18 who completed the CoQ10 intervention (11 patients reported myalgia at inclusion), and 17 who completed the placebo intervention (8 patients reported myalgia at inclusion). An increase was seen in plasma CoQ10 in the CoQ10 supplementation group, indicating adherence to the intervention (Table 1). None reported side effects or harm related to the intervention.

### 4.2. Muscle Analysis

**Figure 2:**
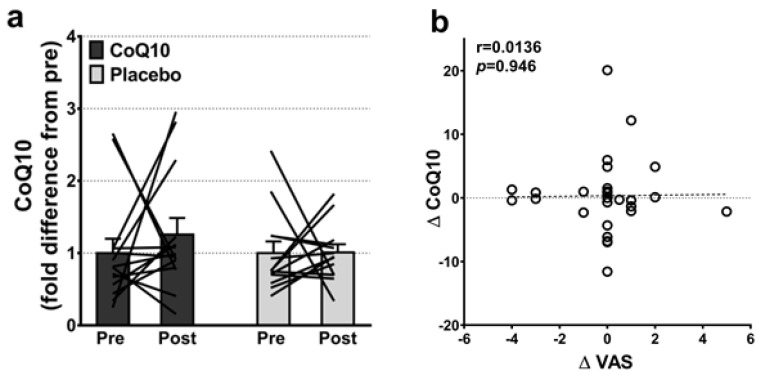
(**a**) Intramuscular CoQ10 levels pre and post CoQ10 and placebo supplementation (n: CoQ10 = 14, placebo = 13). The lines shows pre and post measurements in each individual. (**b**) Correlation between changes in individual pain perception (ΔVAS) and individual changes in intramuscular CoQ10 levels (ΔCoQ10) (n = 27). Error bars on “Pre” are SEM of fold difference from the mean.

**Figure 3:**
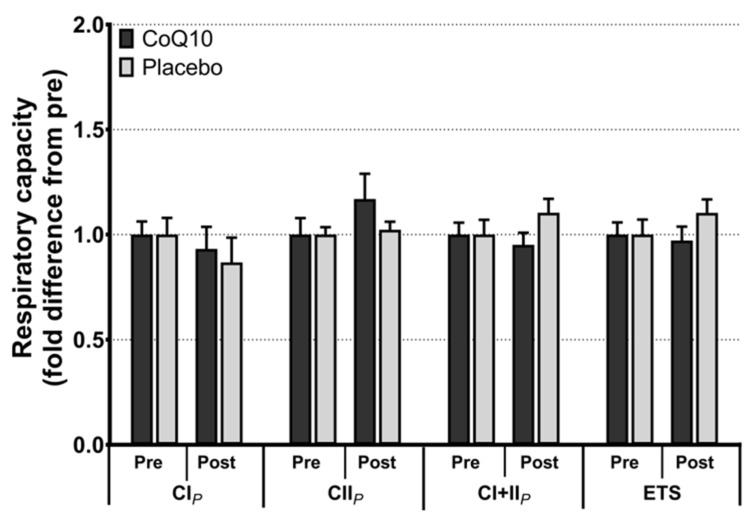
Maximal mitochondrial respiratory capacity with electron flow through complex I (CI*_P_*), complex II (CII*_P_*), complex I + II (CI + II*_P_*), and maximal uncoupled respiration (electron transport system capacity, ETS). Data is expressed as fold difference from pre (mean ± SEM, n: CoQ10: 15, placebo: 14). Error bars on “Pre” bars are SEM of fold difference from the mean.

**Figure 4:**
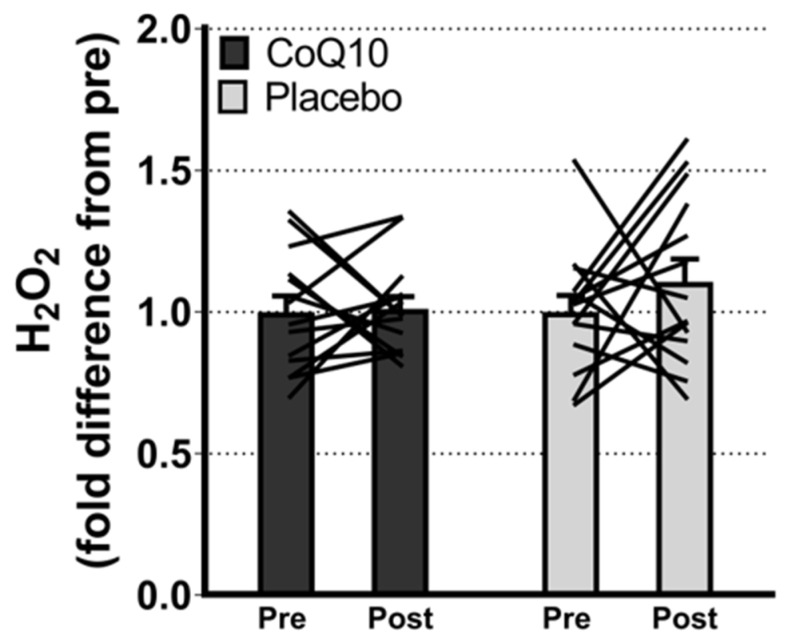
Maximal ROS (H_2_O_2_) production with convergent electron flow through mitochondrial complex I and II. Data are expressed as fold difference from pre (mean ± SEM, CoQ10: 14, placebo: 14). The lines shows pre and post measurements in each individual. ROS: Reactive oxygen species. Error bars on “Pre” bars are SEM of fold difference from the mean.

Muscle CoQ10 levels were unchanged by CoQ10 supplementation and individual changes in muscle CoQ10 levels did not correlate with individual changes in the intensity of myalgia (VAS) (r = 0.0136; *p* = 0.948, Figure 2). Mitochondrial capacity for oxidative phosphorylation did not increase following CoQ10 supplementation (Figure 3), and ROS production (Figure 4) and CS activity (marker of mitochondrial content) were unchanged (CoQ10: 131 ± 8 to 137 ± 8 and placebo: 126 ± 11 to 127 ± 11 µmol·g^−1^·min^−1^, respectively). Mitochondrial respiratory capacity normalized to mitochondrial content (proxy for intrinsic mitochondrial function) was unchanged following the intervention (Supplementary Figure S1), and so was the protein content of Catalase, MnSOD, Caspase-3, and Bcl-2 (Supplementary Figure S2). VEGF was increased following CoQ10 supplementation, but there was no group by time interaction (Supplementary Figure S2).

## 5. Discussion

Contrary to our hypothesis, we found no effects of oral CoQ10 supplementation on muscle CoQ10 levels or mitochondrial function, and individual changes in muscle CoQ10 levels did not correlate with individual changes in intensity of myalgia. We saw an increase in plasma CoQ10 concentration in the CoQ10 group, indicating adherence to the supplementation protocol.

Randomized placebo-controlled trials are considered the gold standard to investigate treatment and intervention effectiveness as they limit the risk of bias due to confounders and placebo effects. Placebo-controlled trials have previously investigated the effects of CoQ10 supplementation on intensity of myalgia [20,21,22,23,24,25,26]. Of these, two reported alleviated myalgia upon CoQ10 supplementation, but none of the studies included the placebo group in the statistical analyses [25,26]. The reported intra- and intergroup differences were solely based on paired and unpaired *t*-tests, and therefore we question the strength of the associations. In contrast, a strength of this study is that all data reported are relative to the placebo group.

CoQ10 is a vitamin like lipophilic cofactor, mainly available from endogenous biosynthesis, but also available from dietary sources. It is estimated that an omnivorous diet provides approximately 5 mg of CoQ10 per day [45]. Secondary deficiency of CoQ10 are well documented in ageing [46,47] and increased request due to intense physical exercise [48] or disease conditions associated with increased oxidative stress [49]. Nutritional supplementation might represent a useful strategy in the latter conditions however CoQ10 is characterized by a very low bioavailability with low levels of dietary CoQ10 being absorbed and as much as 95% being eliminated with feces [50]. Notably, beside general absorption issues in the digestive tract, selective absorption by different tissues and intracellular translocation remain poorly understood. In fact, while it is generally assumed that CoQ10 is absorbed by passive diffusion and membrane recycling, differences in endogenous tissue and subcellular content as well as absorption rate are reported [51]. At the subcellular level, the highest CoQ content is found in mitochondria and the tissues most rich in CoQ are cardiac, skeletal muscle, and liver, where mitochondria are abundant [52]. Notably despite the large amounts and the critical role of the cofactor in these tissues, they appear particularly refractory to exogenous CoQ uptake compared to other tissues such as blood, spleen and liver [51].

We found no changes in muscle CoQ10 levels after eight weeks supplementation with 400 mg CoQ10 daily. The CoQ10 supplementation was divided into two doses of 200 mg to be ingested with a meal to ensure a high absorption, since CoQ10 is absorbed with lipids [15]. With an elimination half-life of ~33 h [15], this supplementation regime should ensure a constant elevation of plasma CoQ10 while allowing for some daily variation in the timing of CoQ10 ingestion. As expected, there was an increase in plasma CoQ10 concentration in the CoQ10 group which confirms adherence to the intervention, and that the CoQ10 was well absorbed. We chose a dose and supplementation period in the high end of the range reported in similar studies (100–600 mg for 30 days to 12 weeks) [20,21,22,23,24,25,26,53,54]. Thus, it is unlikely that our null result is explained by inadequate doses of CoQ10 in the supplementation period.

To our knowledge, only two studies have previously investigated if CoQ10 supplementation increase muscle CoQ10 levels, both in young, statin-naïve adults. In line with our results, one of the studies found unchanged muscle CoQ10 levels but increased plasma CoQ10 levels after four weeks of supplementation with 150 mg CoQ10 daily [55], and the other found a tendency towards an increased muscle CoQ10 after 14 days of daily supplementation with 100 mg CoQ10 [56]. A recent study reported an increase in muscle CoQ10 after four weeks of supplementation [57]. The discrepancies between the human studies might be explained by the bioavailability of the different CoQ10 formulations used. Studies in rodents have revealed conflicting results on the ability of CoQ10 supplementation to increase muscle CoQ10 levels [58,59], and it has been suggested that combining CoQ10 with exercise might improve the absorption of CoQ10 [59,60], which needs further investigation. Since the main coenzyme Q homologue in rodents is CoQ9 [61], as opposed to CoQ10 in humans [62], a direct comparison between animal studies and this study is difficult. The supplementation period of eight weeks in our study was longer than previous studies that measured muscle CoQ10 levels (2–4 weeks). With elevated plasma CoQ10 levels, an increased muscle CoQ10 level was expected, since CoQ10 due to its lipophilic nature should be able to cross the lipid bilayers and enter most cells and mitochondria. In humans, CoQ10 is distributed in most tissues with the majority located in tissues with a high energy turnover and a large amount of mitochondria [15]. It is possible that a longer supplementation period is necessary to achieve an increase in muscle and CoQ10 levels, or the uptake could be limited at the mitochondrial level in statin users. Further research is needed on this topic.

We had hypothesized that mitochondrial respiratory capacity would increase and mitochondrial ROS production would decrease after CoQ10 supplementation as a result of increased muscle CoQ10 levels. Thus, the unchanged muscle CoQ10 levels may explain the unchanged mitochondrial respiratory function and ROS production.

The hypothesis that links statins to reduced CoQ10 synthesis, mitochondrial dysfunction, and myalgia is biologically plausible, but since it arose from case reports in the early 1990s [63,64], it has not been confirmed in any large randomized controlled trials. While some studies have associated statin therapy and statin-induced myalgia with altered mitochondrial function [65,66,67] and others have found reduced muscle CoQ10 in statin users [9,65], an association between impaired mitochondrial function reduced muscle CoQ10 levels, and statin-induced myalgia has still not been confirmed in the literature. This study adds doubt to the proposed benefits of CoQ10 supplementation on statin-induced myalgia, and to whether the mechanism underlying statin-induced myalgia involves reduced muscle CoQ10 levels and impaired mitochondrial function as a consequence. Further research is warranted to elucidate the etiology of statin-induced myalgia and to develop strategies to prevent or alleviate statin-induced myalgia.

Limitations: Our study included statin users who reported none to moderate myalgia. We cannot exclude the possibility that individuals with more severe cases of statin-induced myalgia or with confirmed muscle CoQ10 deficiency, could have beneficial effects of CoQ10 supplementation. However, the subjects included in this study represent people living with statins in the general population. We assessed the intensity of myalgia using VAS, which is an effective and commonly used non-invasive tool to assess intra-individual changes in the intensity of myalgia, but not sensitive enough to quantify small changes in myalgia. Finally, our study design did not allow us to investigate if the myalgia was statin related. For this, we should have established a wash-out period where statin treatment was stopped, as done in Derosa et al. [68]. Gender is not equally distributed in the groups, which could have an impact on the results. We adapted the HPLC-method to measure CoQ10 from a previous study [42], and our method only measures CoQ10 and not its reduced form, “CoQH2”, also known as ubiquinol. However, since CoQ10 content in muscle biopsies is mainly in its ubiquinone form, the data discussed are representative of CoQ10 muscle status and its modulation through dietary intervention.

## 6. Conclusions

We report no effects of oral CoQ10 supplementation on mitochondrial function or muscle CoQ10 levels in statin users with and without myalgia. In statin users with mild to moderate myalgia, individual changes in muscle CoQ10 levels were not correlated with individual changes in the intensity of myalgia. Further studies are warranted to develop strategies to alleviate myalgia for statins users.

## Figures and Tables

**Table 1:** Patient characteristics.

	CoQ10		Placebo	
	Pre	Post	Pre	Post
N (male/female)	18 (14/4)		17 (8/9)	
Age (years)	62 ± 1	-	64 ± 2	-
Body mass index (kg∙m^−2^)	28 ± 1	28 ± 1	29 ± 1	29 ± 1
VO_2_max (mL O_2_∙min^−^^1^ ∙kg^−^^1^)	30 ± 1	30 ± 2	27 ± 1	27 ± 1
Myalgia (VAS, cm)	2.4 ± 0.7	2.2 ± 0.6	1.4 ± 0.5	1.7 ± 0.6
Plasma creatine kinase (μ·L^−1^)	119 ± 15	153 ± 53	92 ± 11	92 ± 9
Total blood cholesterol (mmol∙L^−1^)	4.2 ± 0.2	4.1 ± 0.2	4.1 ± 0.2	4.3 ± 0.3
Blood LDL-c (mmol∙L^−1^)	2.6 ± 0.1	2.5 ± 0.2	2.4 ± 0.2	2.7 ± 0.3
Plasma CoQ10 (nmol∙L^−1^)	545 ± 46	2627 ± 405 *	468 ± 41	518 ± 59
Plasma CoQ10/chol (nmol ∙ mmol^−1^)	133 ± 11	650 ± 90 *	109 ± 9	118 ± 10

Patient characteristics. * *p* < 0.001 & Group by time interaction, Abbreviations: chol: total plasma cholesterol, LDL-c: Low density lipoprotein, VO2max: maximal oxygen consumption, VAS: visual analogue scale, cholesterol, Data are mean ± SD.

## Data Availability

Some or all datasets generated during and/or analysed during the current study are not publicly available but are available from the corresponding author on reasonable request.

## Supplementary Materials

The following supporting information can be downloaded at: www.mdpi.com/article/10.3390/antiox11091698/s1. Figure S1 Mitochondrial respiratory capacity normalized to marker of mitochondrial content. Figure S2 Muscle content of proteins.
