# Supplementary material for: Coenzyme Q10 Supplementation in Statin Treated Patients: A Double-Blinded Randomized Placebo-Controlled Trial
Source: Antioxidants (Basel). 2022 Aug 29;11(9):1698. doi: 10.3390/antiox11091698 (PMC9495827; doi:10.3390/antiox11091698)
Supplement: Supplementary file 1 [file antioxidants-11-01698-s001.zip › antioxidants-1865248-supplementary.pdf]

Supplemental material

Methods

Western blotting

Western blotting of catalase, manganese superoxide dismutase (mnSOD), vascular endothelial growth factor (VEGF), caspase-3, and B-cell lymphoma 2 (bcl-2) were performed. In brief, freeze-dried muscle tissue homogenates were loaded onto Stain-Free polyacrylamide sodium dodecyl sulphate (SDS) gels (Criterion, Bio-rad, Copenhagen, Denmark) and transferred to polyvinylidene fluoride (PVDF) membranes (Bio-Rad, Copenhagen, Denmark). The membranes were blocked in 5% milk diluted in Tris-buffered saline and 0.05% Tween 20 or Phosphate buffered saline at room temperature and incubated in primary antibody overnight in 4 ^{o}C: anti-mnSOD (06-984 Millipore), anti-catalase (RnDSystems), anti-caspase-3 (#9662, Cell Signaling Technology), anti-Bcl-2 (#4223 Cell Signalling Technology), and anti-VEGF (ab46154, Abcam, Cambridge, UK). Secondary antibodies were polyclonal goat anti-rabbit horseradish peroxidase conjugated (DAKO, Glostrup, Denmark). The samples were loaded in a randomized order to distribute samples from the placebo and CoQ10 groups evenly, but pre and post samples were adjacently loaded. All samples were quantified relative to an average of three calibrator-samples loaded to adjust for inter-gel variances to compare the samples loaded on different gels.

Figures

Supplementary Figure S1 Mitochondrial respiratory capacity normalized to marker of mitochondrial content

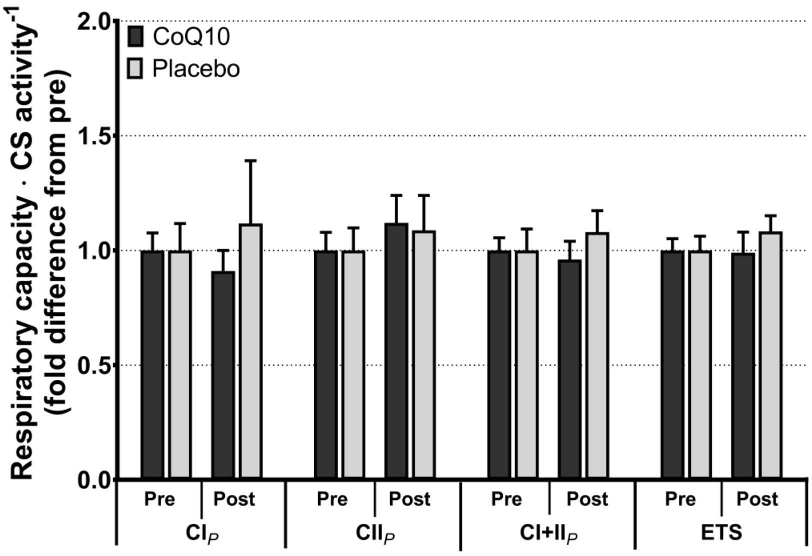

Mitochondrial intrinsic respiratory capacities. Mitochondrial respiratory capacities normalized to CS activity as a biomarker of mitochondrial content. Data is expressed as fold difference from pre (mean ± SEM, n: CoQ10: 14, placebo: 8). Error bars on “Pre” bars are SEM of fold difference from the mean.

Supplementary Figure S2 Muscle content of proteins

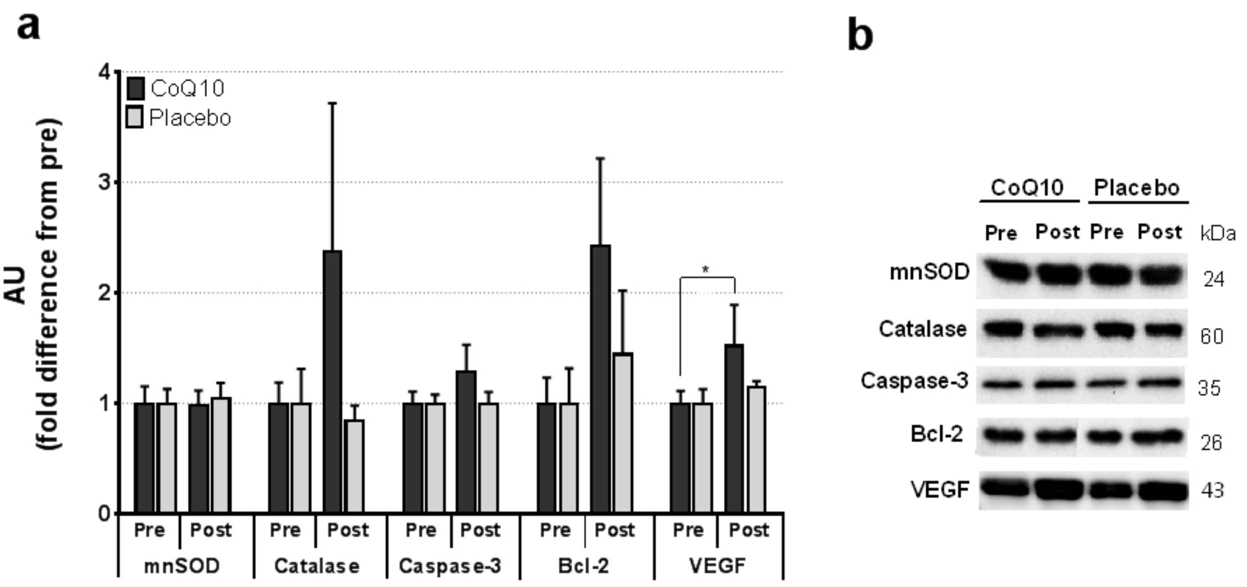

a: Abundance of proteins measured by western blotting. Post intervention samples are expressed as fold differences from pre (mean ± SEM, n: CoQ10: 11, placebo: 8). AU: Arbitrary units. *post different from pre within group, P<0.05. Abbreviations: Bcl-2: B-cell lymphoma 2, VEGF: vascular endothelial growth factor. **b**: Representative blots from one subject per group are presented. Error bars on "Pre" bars are SEM of fold difference from the mean.
